# Point-of-Care Virtual Planning and 3D Printing in Facial Trauma: A 10-Year Experience at a Single Institution

**DOI:** 10.3390/jcm14082788

**Published:** 2025-04-17

**Authors:** Sara M. Hussein, Doga Kuruoglu, Jonathan M. Morris, Victoria A. Sears, Abdallah A. Shehab, Waleed Gibreel, Basel A. Sharaf

**Affiliations:** 1 Division of Plastic and Reconstructive Surgery, Department of Surgery, Mayo Clinic, Rochester, MN 55905, USA; hussein.sara@mayo.edu (S.M.H.); kuruoglu.doga@mayo.edu (D.K.); gibreel.waleed@mayo.edu (W.G.); 2 Neural Engineering and Precision Surgery Laboratories, Mayo Clinic, Rochester, MN 55905, USA; 3 Division of Neuroradiology, Department of Radiology, Mayo Clinic, Rochester, MN 55905, USA; 4 Anatomic Modeling Lab, Department of Radiology, Mayo Clinic, Rochester, MN 55905, USA

**Keywords:** virtual surgical planning, three-dimensional printing, facial trauma, mandibular fractures, maxillofacial fractures, point of care

## Abstract

**Background**: Despite increased adoption of virtual surgical planning (VSP) in various craniofacial indications, the incorporation of VSP/3DP into facial trauma care remains limited. Therefore, Expedited Preoperative Point of Care for Fracture Reduction to Normalized Anatomy and 3DP to Improve Surgical Outcomes (EPPOCRATIS) was introduced in 2021. This study evaluates our experience with EPPORATIS in craniomaxillofacial trauma over 10 years. **Methods**: A retrospective review of patients who underwent facial trauma repair between September 2014 and September 2024 was conducted. For each VSP/3DP case, a patient with similar facial trauma patterns, who was treated without VSP, was selected. Evaluation metrics included operative time, blood loss, length of stay, complication rates, and fracture reduction accuracy through 3D heatmap analyses. Operative metrics were normalized by implant (i.e., fracture plates and screws) count to account for fracture complexity. A value of *p* < 0.05 was deemed statistically significant. **Results**: The VSP group presented with more complex injuries and higher involvement of various surgical specialties (*p* < 0.5) and demonstrated longer operative times (*p* < 0.03). Although the difference was not statistically significant (*p* = 0.4), when adjusted for implant count, the VSP group had shorter operative times (median: 15.4 vs. 19.3 min/implant) and reduced blood loss compared to non-VSP cases (median: 3.4 mL/implant vs. 4.2 mL/implant). Complications, revision rates, and length of stay showed no significant differences. **Conclusions:** The use of VSP/3DP (EPPOCRATIS) in craniomaxillofacial trauma reconstruction demonstrated operative efficiency and accurate fracture reduction in complex cases. Further studies are needed to examine the feasibility and cost-effectiveness of point-of-care VSP/3DP in trauma centers.

## 1. Introduction

Facial trauma demands precise reconstructive procedures to restore function as well as the aesthetic form of the face [1]. Virtual surgical planning (VSP) and three-dimensional printing (3DP) have transformed the way reconstructive surgery is performed by creating accurate physical models of the 3D spatial relationships of craniomaxillofacial structures. Currently, with the assistance of computer-assisted design (CAD), surgeons can conduct virtual surgeries and generate templates and cutting guides, enabling the accurate recreation of surgical plans in the operating room [2]. Consequently, VSP has been widely adopted for surgical procedures, including oncological resections, orthognathic surgery, free flap transfer, facial gender-affirming surgery, and facial allograft transplantation [3,4,5,6,7,8], which underlines VSP’s effectiveness in maximizing surgical precision and outcomes.

Comparatively, VSP adoption in facial trauma reconstruction has been rather limited. Recognizing this gap, an Expedited Preoperative Point of Care for Fracture Reduction to Normalized Anatomy and Three-dimensional Printing (EPPOCRATIS) system has been developed in-house by the senior author (B.A.S.) and previously been published [9,10]. This approach was designed to bring the benefits of VSP to facial trauma repair. Thus, it allows a streamlined workflow to meet the demands of urgent trauma care [11].

The aim of this study is to compare our experience with point-of-care VSP/3DP (EPPOCRATIS) and conventional (non-VSP) treatment of facial fractures at a single academic institution. Specifically, we evaluated operative variables such as time, blood loss, and postoperative 3D bony alignment over a 10-year period, comparing the two approaches.

## 2. Materials and Methods

After approval by our Institutional Review Board (IRB #: 23-010055) on 22 December 2023, a retrospective review of patients who underwent surgical repair of facial fractures by the senior author (B.A.S.) at our tertiary care center between September 2014 and September 2024 was performed.

### 2.1. Patient Selection

The inclusion criteria encompassed all primary facial trauma patients who underwent open reduction and internal fixation, either with or without VSP, with no age restriction applied. Patients with pre- and postoperative facial computed tomography (CT) scans and at least 1 week of follow-up were included. Long-term follow-up in facial trauma patients, although desirable, is difficult to achieve. Closed fracture reduction cases, lack of CT imaging, or follow-up were our exclusion criteria. A cohort of patients who underwent conventional fracture repair techniques at our institution (No VSP/3DP) was matched to patients who underwent operative management with VSP assistance, ensuring demographic similarities and comparable fracture patterns. These matching parameters included age, gender, race, BMI, medical comorbidities like hypertension (HTN) and diabetes (DM), smoking habits, and alcohol or drug use. Patients were classified as active smokers if they reported smoking any cigarettes within 30 days prior to surgery. Former smokers were defined as patients who did not smoke any cigarettes within the 30 days prior to surgery but had a history of smoking more than 100 cigarettes in their lifetime. This matching process aimed to maintain objectivity in evaluating the differences in operative metrics between the two approaches.

### 2.2. Data Collection

Key clinical parameters were then collected to provide a comprehensive assessment of the patients included. Epidemiological factors included the mechanism of injury, such as motor vehicle accidents (MVAs), assaults, or falls, trauma settings (home, work, or street), along with concomitant trauma sites. Additionally, clinical data captured fracture patterns, including naso-orbital ethmoid (NOE) fractures, orbital fractures, zygomaticomaxillary complex (ZMC), mandibular fractures, or panfacial fractures. We recorded the fracture severity, such as displacement and comminution, based on the radiological assessment and physical examination in the patients’ records. Operative metrics included the timing from injury to surgery, operative time, use of VSP and its date of consultation, implants (type and count), and specific surgical approaches, such as intraoral, subtarsal, or bicoronal incisions. Postoperative outcomes, including the length of hospital stay, complication rates (infection, discomfort, implant complications, bone malunion, or nerve injury), and necessity of secondary surgeries, were comprehensively evaluated. The CT scans were acquired in the emergency setting using one of several Siemens Healthineers CT scanners (Siemens Medical Solutions Inc., Malvern, PA, USA). All scans followed a standardized trauma protocol for facial imaging, which includes non-contrast acquisition from the vertex to the mandible in a spiral, craniocaudal direction; 120 kVp, 350 mAs, 128 × 0.75 mm collimation, CTDI of approximately 50 mGy; field of view 250 mm. Images were reconstructed at 0.6 mm and 0.75 mm slice thicknesses using both bone and soft tissue kernels, with sagittal and coronal reformats. Digital Imaging and Communications in Medicine (DICOM) files of the patients were then exported for 3D reconstruction. Preoperative and postoperative CT scans were segmented and analyzed using Materialise Mimics software v25.0 (Leuven, Belgium) [12]. When possible, the Iterative Metal Artifact Reduction (IMAR) protocol was applied during image acquisition and segmentation to minimize artifacts caused by dental metals and postoperative implants [13].

#### 2.2.1. Three-Dimensional (3D) Segmentation Workflow

**Figure 1:**
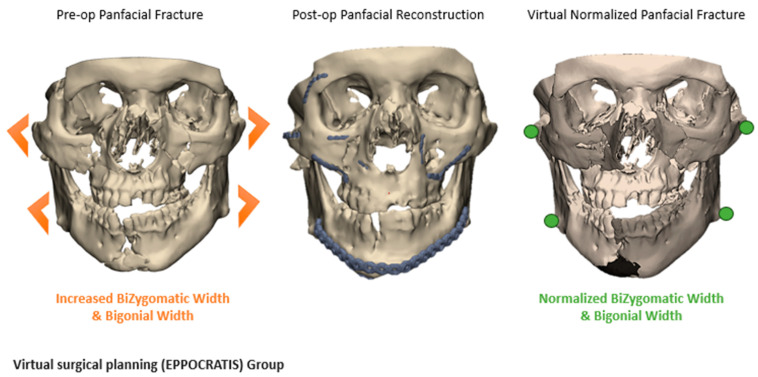
Panfacial fracture reconstruction using VSP (EPPOCRATIS Protocol [10]).

**Figure 2:**
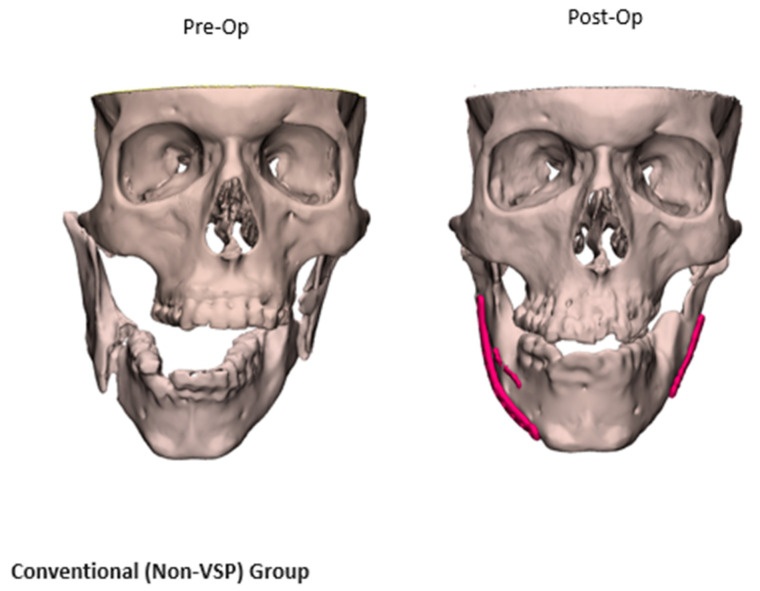
Facial fracture reconstruction in the non-VSP group.

The workflow for VSP included the following steps: (1) Preoperative CT scan acquisition and segmentation. For patients in the VSP group, preoperative and postoperative CT scans were segmented using Mimics software as previously described [9]. Segmentation of anatomical structures was standardized using the built-in bone thresholding feature in Materialise Mimics, at a Hounsfield unit limit of 226. (2) After segmentation of the displaced fracture segments. Virtual reduction and alignment in Mimics was performed by experienced biomedical engineers at our Anatomic Modeling Unit (AMU). To ensure consistency across cases, smoothing algorithms were minimized to prevent loss of anatomical details. Achieving the best anatomical bony alignment was performed in multiple CT planes (axial, coronal, and sagittal), ensuring optimal anatomical repositioning. This often required input from the senior author (B.A.S). (3) Generation of 3D-printed models of the virtually reconstructed anatomical structures (Figure 1), allowing surgeons to preoperatively bend the fracture plates and plan the fixation plates’ (implants’) placement. Manual refinement was necessary in the postoperative 3D models after isolating the metal fixation plates from the underlying bone. For mandibular fractures, the occlusal alignment was optimized based on tooth-bearing bony segment alignment. The mandibular condyles were symmetrically seated within the glenoid fossae, and the positioning was verified in 3 CT planes on Mimics, including slice-by-slice editing and inspection in all these 3 anatomical planes. Final occlusal alignment was achieved intraoperatively using maxillo-mandibular fixation. In orbital and ZMC fractures, mirror imaging of the unaffected side was used to reconstruct the affected orbital walls or establish symmetry of the ZMC fracture segments [9]. A similar 3D segmentation process was carried out for the non-VSP group; while virtual planning was not performed, both preoperative and postoperative CT scans were obtained and compared using the heatmap feature (Figure 2).

#### 2.2.2. Comparative Analysis of VSP and Non-VSP Groups

Consequently, the surface-based registration algorithm was used to superimpose the 3D models together using an iterative closest point (ICP), focusing on stable cranial reference areas that are unaffected by fracture. Comparative analyses were performed on both the VSP and non-VSP groups, evaluating fracture alignment and assessing the impact of VSP or conventional methods on fracture alignment precision and outcomes. Heatmap analyses were utilized to visualize and quantify discrepancies in bone alignment between the 3D models. Furthermore, fracture reduction accuracy was evaluated using the root mean square error (RMSE) through these generated heatmaps from pre- and postoperative 3D models. Thus, RMSE higher values, which represent the average distance in millimeters between corresponding surface points across two models, indicated greater discrepancies between the before and after 3D models [14]. The heatmaps were generated in 3-matic software and displayed as a standardized color scale, representing both the direction and the magnitude of bone positioning. Blue (minimum deviation) indicated areas where the bone was displaced outward, away from the midline or reference surface, while red (maximum deviation) represented regions of depression or inward displacement toward the center of the anatomical structure. In summary, our primary outcomes focused on operative workflow efficiency, bone alignment, and complication rates, while our secondary outcomes analyzed the need for any revision surgeries or subsequent procedures, as well as any patient-reported outcomes included in the charts.

### 2.3. Statistical Analysis

Descriptive statistics were calculated for both groups, representing individual patients’ characteristics. Normally distributed numerical data were presented using mean ± standard deviation (SD), whereas non-normally distributed numerical data were presented using median and interquartile range (IQR). The independent *t*-test and the Wilcoxon Rank Sum (Mann–Whitney U) test were used to compare normally and non-normally distributed continuous variables, respectively. The chi-square and Fisher exact tests were used to compare categorical variables such as complication rates and reoperation rates. Statistical analysis was performed using R Studio (v4.5), and visualizations were performed using Python (v3.8) with the Matplot library for boxplots. A value of *p* < 0.05 was deemed statistically significant.

## 3. Results

**Figure 3:**
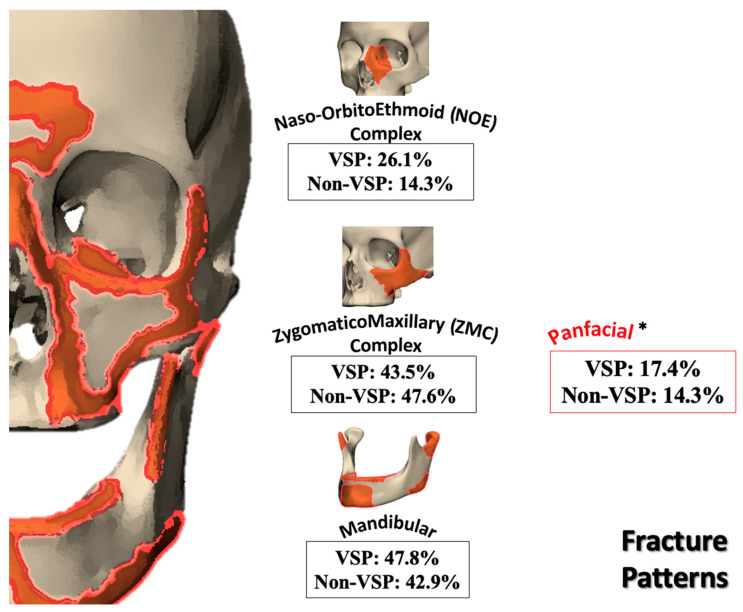
Distribution of fracture patterns in VSP and non-VSP groups. * Panfacial denoted fractures that involve multiple facial subunits, including Le Fort classification and extend across the midface and the mandible.

Of the 242 fracture repairs treated by the senior author (B.A.S.) over a 10-year period, 44 cases met the inclusion criteria and were incorporated into the final analysis. A total of 23 patients underwent facial fracture repair with the VSP/3DP integration. A matched cohort of 21 patients who underwent repair using conventional techniques (non-VSP) was selected, as summarized in Table 1. To this end, the fracture patterns observed in our study were rarely isolated, with severe trauma involving multiple facial regions. Each fracture was individually categorized into multiple fracture categories, resulting in a cumulative percentage that surpassed 100% (Figure 3).

### 3.1. Demographics and Patients’ Characteristics

Of 44 patients, the majority were males (*n* = 36, 81.8%) and white (38, 86.4%). The average age of the study cohort was 35.6 years (SD: 16.9 years), with a mean BMI of 26.14 kg/m^2^ (SD: 6.5 kg/m^2^) (Table 1). In addition to plastic and reconstructive surgery, other surgical specialties involved in the procedures included oculoplastic surgery (22.7%), otolaryngology (15.9%), neurosurgery (13.6%), and orthopedic surgery (11.4%). Thus, oculoplastic surgery and otolaryngology were significantly more involved in the VSP cases (*p* = 0.05 and *p* = 0.01, respectively), as shown in Table 2. Regarding the matched comorbidities, hypertension was present in 6.8% of patients, while diabetes was noted in 11.4% and anticoagulants use was reported in 9.1% of the cohort. Lifestyle factors included alcohol use in 31.8% of patients and smoking in 34.1%, with 86.7% of smokers being current smokers and 13.3% being former smokers. Additionally, drug use was documented in 15.9% of patients. These baseline characteristics were similar between the VSP and non-VSP groups. Nevertheless, motor vehicle accidents (MVAs) were more frequently the mechanism of injury in the VSP group compared to the non-VSP group (43.5% vs. 19%, *p* = 0.09). Although this difference was not statistically significant, the trend indicated that high-energy, complex injuries likely necessitated VSP/3DP to optimize surgical outcomes and enhance the communication between the surgical teams.

### 3.2. Injury Mechanisms and Fracture Patterns

**Figure 4:**
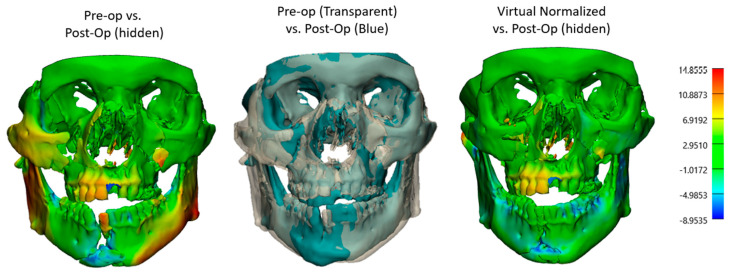
Heatmap analyses and 3D overlays, comparing the preoperative and virtually reduced models to the postoperative 3D model in millimeters. On the right 3D heatmap, a more uniform green color indicates 1–2 mm discrepancy between the planned and postop 3D CT.

Fracture patterns were varied (Table 2), with zygomaticomaxillary complex (ZMC) fractures and mandibular fractures being the most common (Figure 3). Fifty-six percent of fractures were bilateral, with varied fracture complexity between the two sides. Mandibular fractures were most observed at the condyles and symphysis regions, each accounting for 47.1% of cases, followed by the body (41.2%) and the rami (23.5%). Mandibular fractures often presented with symptoms such as pain, misaligned teeth, loose or missing teeth (53.8%), and abnormal jaw movement (38.5%). On the other side, orbital floor fractures were observed in 94.7% of cases with orbital injuries, whether isolated or associated with other fracture patterns, followed by orbital walls (52.6%) and the orbital roof (26.3%). Diplopia was the most common presenting symptom with orbital involvement (56.3%), followed by enophthalmos or globe malposition (18.8%). Despite matching the fracture patterns, the VSP group displayed a greater incidence of displaced and comminuted fractures, as further quantified in the 3D overlays and heatmap analyses (Figure 1 and Figure 4).

**Figure 5:**
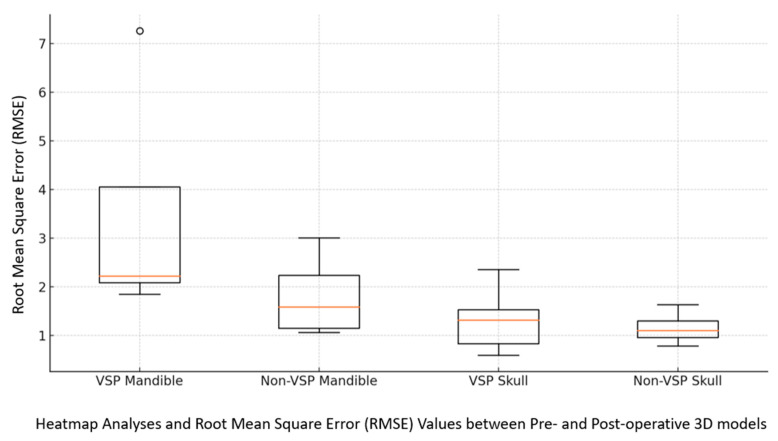
The calculated RMSE of the heatmaps between the 3D models of both VSP and non-VSP groups. The circle represents an outlier value in the dataset (VSP Mandible).

Moreover, when RMSE values between the pre- and postop 3D models of both the VSP and non-VSP groups were assessed, the VSP group showed remarkable initial complexity and more displacement in their preoperative models (Figure 4 and Figure 5). In particular, the median RMSE values were higher in the VSP mandibles (VSP RMSE = 2.2 mm vs. non-VSP RMSE = 1.6 mm, *p* > 0.05), whereas upper and midface fractures (skull) exhibited lower variability in both groups (VSP RMSE = 1.3 mm vs. non-VSP RMSE = 1.1 mm, *p* > 0.05). This can be explained by the inherent anatomical stability of the midface compared to the mandible. Additionally, color-coded heatmaps in Figure 4 visualized regional discrepancies in different facial subunits preoperatively. Comparing the planned and postoperative 3D CTs demonstrated improved symmetry and color uniformity on the heatmap following fracture reconstruction (Figure 4), indicating greater concordance between the virtual surgical plan and the postoperative outcome.

### 3.3. VSP vs. Non-VSP Groups’ Characteristics

**Figure 6:**
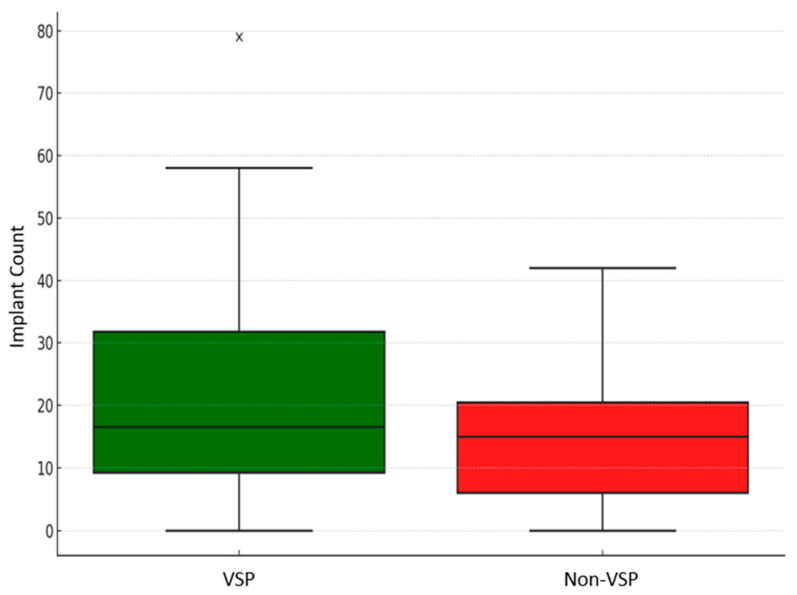
Implant count comparison between the VSP and non-VSP groups. The cross mark represents an outlier value in the dataset (VSP).

Although tremendous efforts were made to match fracture patterns between the VSP and non-VSP groups, the VSP group showed markedly greater severity in both fracture patterns and reconstruction complexity (Figure 3 and Figure 5). Of the patients in both groups, 82.6% in the VSP group and 95.2% in the non-VSP group presented with mild TBI. Moderate and severe TBI were more prevalent in the VSP group (Table 2). The American Society of Anesthesiologists (ASA) status of the VSP group also includes more patients with severe systemic illnesses (ASA 3 and ASA 4). This indicated that VSP is mostly utilized for complicated facial trauma cases that need comprehensive surgical planning. The non-VSP group, on the other hand, generally comprised healthier patients (ASA 1). Furthermore, higher implant counts confirmed this perceived surgical complexity in comparison (Figure 6). Documented in the patient’s operative note, the implants accounted for both the fracture fixation plates and the screws utilized.

### 3.4. VSP vs. Non-VSP Groups’ Characteristics

**Figure 7:**
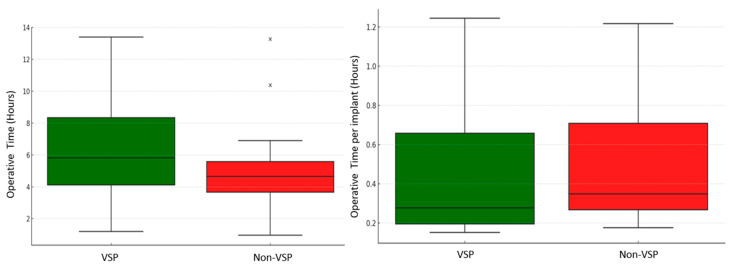
Comparison of operative times between the VSP and non-VSP groups, before and after adjustment for implant count. The cross marks represent outlier values in the dataset (Non-VSP).

**Figure 8:**
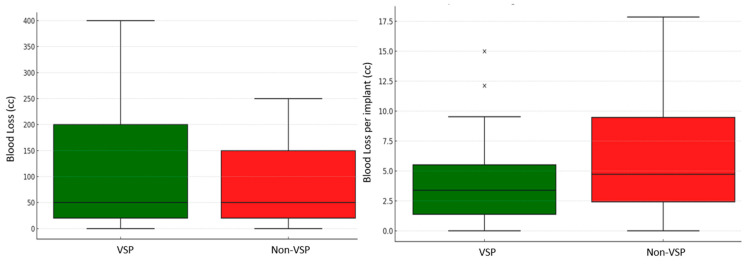
Comparison of blood loss between the VSP and non-VSP groups, before and after adjustment for implant count. The cross marks represent outlier values in the dataset (VSP).

The median total anesthesia time for the VSP group was 5.8 h (IQR = 4.1–8.3 h), and it was 4.6 h (IQR = 3.7–5.6 h) for the non-VSP group. Similarly, the median total procedure time for the VSP group was 3.8 h (IQR = 2.5–5.6 h), and it was 2.7 h (IQR = 2.1–3.6 h) for the non-VSP group. Given the previously mentioned complexity of the VSP group, this significant trend toward a longer operative time was expected (*p* = 0.03). To account for fracture complexity, the surgical metrics were normalized by the number of implants used in each case (Figure 6). This adjustment provided a balanced comparison of surgical efficiency between the VSP and non-VSP groups. When normalized by the number of implants placed, these differences were further contextualized, indicating that these prolonged operative times among the VSP group were proportional to the surgical complexity (Table 3). Consequently, the median anesthesia time per implant showed a notable reduction in the VSP group compared to the non-VSP group (15.4 min, IQR = 11.3–33.8 vs. 19.3 min, IQR = 15.7–31.4; *p* = 0.4). Similarly, the median procedure time per implant was slightly lower in the VSP group compared to the non-VSP group (10.5 min, IQR = 6.7–22.5 vs. 10.9 min, IQR = 9–17.7; *p* = 0.5) (Figure 7). Although these differences did not show statistical significance, the VSP group demonstrated a promising trend toward reduced per-implant operative times. Additionally, the mean blood loss was less in the VSP group with 3.4 mL/implant, while that in the non-VSP group was 4.1 mL/implant (*p* = 0.7), indicating less tissue manipulation (Figure 8). The median length of hospital stay was 1 day for both groups, spanning from 0 to 21 days for the VSP and 0 to 16 days for non-VSP (*p* = 0.7). The follow-up period ranged from 9 to 1622 days, with medians of 65 (IQR = 34–169) days and 43 (IQR = 30–74) days for the VSP and non-VSP groups, respectively. No significant differences were observed in the complication rates between the two groups. Of the 23 VSP cases and 21 non-VSP cases, 1 case from each category required subsequent intervention due to complications. The VSP case presented with hardware infection involving three screws with pseudo-capsule formation in the chin region, necessitating hardware removal and debridement. This complication was attributed to a continued smoking status. The non-VSP case, on the other hand, presented with phlegmon and fibrous encapsulation around the orbital floor implant, requiring implant removal. These reported complications did not exhibit any notable difference in severity between the two groups, despite the VSP group having initially more complex facial fractures from high-impact trauma (Table 2).

### 3.5. Fracture Reduction Accuracy

**Figure 9:**
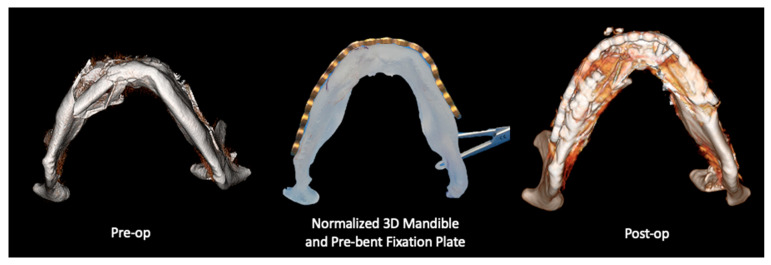
Preoperative, virtually planned reconstruction 3D printed model with a pre-bent fixation plate, and the postoperative mandibular 3D models.

A comparative analysis of the preoperative, virtual reduced, and postoperative 3D models was conducted to assess the bone alignment and reduction accuracy of the facial fractures. The virtual planned model and the resulting physical 3D-printed model served as a base for pre-bending the fixation plane for intraoperative alignment with the bone, demonstrated in Figure 9.

**Figure 10:**
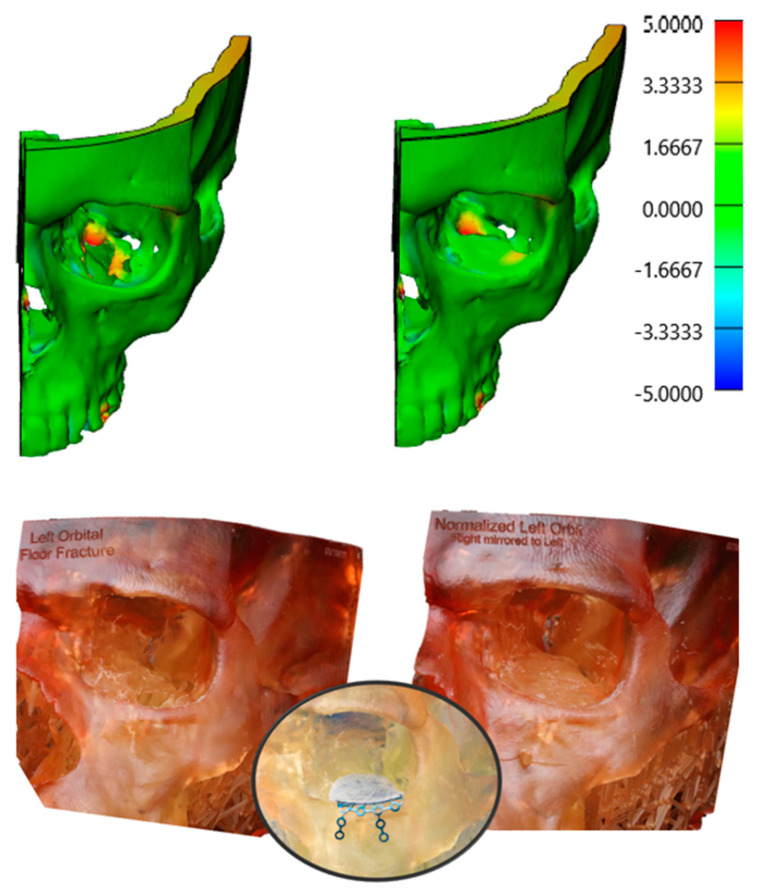
Heatmap analyses showing two comparisons: (1) preoperative anatomy versus postoperative orbital 3D model, and (2) virtually planned (normalized) reconstruction vs. postoperative orbital 3D models. The color scale indicates the surface deviation in millimeters (mm). Additionally, a pre-bent Medpor (porous polyethylene) orbital plate was perfectly aligned with the bony contour to the reconstructed orbital floor on the 3D printed models.

Furthermore, pairwise heatmap analyses between these models were assessed (Figure 4 and Figure 5), showing neutralization of colors, minimizing the deviations, and enabling precise anatomical restoration of the normalized bone contour (Figure 10).

## 4. Discussion

This study highlights that although the VSP group had considerably more complicated fracture patterns, more severe systemic disease (higher ASA status), and multiple surgical specialties involved, the heatmap analyses showed that the median pre- and postoperative RMSEs in the VSP and non-VSP groups were similar. The VSP group also demonstrated greater surgical efficiency when normalized for implant count, with shorter operative times per implant (median: 15.4 vs. 19.3 min/implant for non-VSP, *p* > 0.05) and reduced blood loss per implant (mean: 3.4 mL/implant for VSP vs. 4.2 mL/implant for non-VSP). Importantly, there were no significant differences in overall complication rates or length of stay (median: 1 day for both groups). These findings demonstrate the value of VSP in managing complex cases, facilitating accurate fracture reduction, and enhancing surgical efficiency despite greater procedural challenges.

Previous studies have highlighted the advantages of VSP and 3DP technologies in various craniofacial indications, particularly in terms of improving surgical accuracy and efficiency through a multidisciplinary approach [9,10]. For example, Moore et al. reported that the use of VSP has been associated with improved alignment and precision in complex maxillectomy reconstruction, leading to better postoperative results [2]. In their study, 23 patients underwent total or partial maxillectomy using the VSP and 3DP, among which no patients experienced lateral rhinotomy (LR) complications, with only one flap failure in the non-LR group [2]. In contrast, fifty percent of the patients in the non-VSP group developed LR-related complications [2]. Therefore, the authors concluded that the VSP integration enhanced the flap utility while reducing the need for external surgical incisions [2]. Additionally, the VSP presented a high accuracy in atrophic edentulous mandible fracture management, with discrepancies below 1.5 mm and favorable symmetry and clinical outcomes [15]. In orthognathic surgery, Ho et al. highlighted the advantage of using simulated 3D models to overcome the 2D aspect of cephalometric analysis and improve the accuracy of maxillomandibular repositioning [16,17]. The authors reported an acceptable range of RMS difference between the 3D simulated models and postsurgical models, with values of 0.6 ± 0.25 for maxilla and 0.85 ± 0.4 for the mandible [16,17]. Our prior research has emphasized this fact, illustrating that despite the meticulously planned virtual model, the adjustment of mandibular occlusion intraoperatively remains the gold standard to guarantee a superior function and aesthetic outcome [9,10]. Similarly, reduced ischemia times and improved bone contact in fibula free flap reconstructions have been reported using computer-aided design and manufacturing (CAD-CAM) [18]. Such adoption of in-house CAD/CAM technology represents a transformative step in head and neck reconstruction. Vranckx et al. also highlighted that insourced VSP reduced the net financial burden compared to outsourced services and ensured high precision in bone flap reconstruction with a 94.7% survival rate [7]. In a related study, Troise et al. conducted a quantitative analysis of mandibular fracture management comparing in-house VSP-assisted versus conventional surgical techniques. Their findings revealed that VSP resulted in more anatomically accurate reductions [19].

Facial fractures can result from a wide range of causes, including motor vehicle accidents and physical assaults [20]. The challenges of limited visibility and procedural inefficiency in facial trauma reconstruction necessitate innovative solutions [9,10]. This advanced approach surpasses conventional methods, offering surgeons the ability to achieve accurate alignment and symmetry, ultimately incorporating other emerging technologies to improve surgical outcomes. For instance, Zavattero et al. demonstrated that the navigation system reduced postoperative diplopia and restored orbital volume compared to traditional approaches in orbital fracture repair [21]. Similarly, Bergeron et al. showed that intraoperative navigation reduced the operative time by 36.1% when treating acute major facial fractures [22]. Our findings add to this body of evidence by specifically focusing on facial trauma cases and demonstrating the measurable impact of VSP on fracture reduction accuracy. Nevertheless, surgeons must make dynamic adjustments to a detailed preoperative plan during surgery to ensure proper dental occlusion and jaw function, which may lead to deviations from the initial plan [9,10].

Notably, there is an ongoing debate in the literature regarding the cost-effectiveness and practicality of implementing VSP widely. Surgeons argue that the high cost and need for specialized training and equipment may limit its accessibility and applicability in everyday clinical practice. Our findings elucidated that there is a significant reduction in operative time per implant count in the VSP group. Additionally, several studies emphasized that the in-house VSP/3DP workflow significantly reduces the reliance on third-party services, lowers the costs, speeds up the production, and promotes a personalized surgical practice [7]. While it is beyond the scope of this study, it is important to acknowledge that although our findings complemented the notion that VSP is cost-effective in highly complicated cases, these technologies depend on institutional factors, such as patient flow, case complexity, and available resources [10]. Facilities with a high volume of complex cases may find VSP to be a valuable investment, offering efficiency and long-term cost savings. However, for institutions with a lower volume of such cases, the return on investment may be less pronounced. Beyond cost savings, VSP offers other advantages that are hard to quantify, such as enhancing patient–surgeon interactions and improving patients’ understanding of the procedure and expected outcomes using 3DP models. In addition, the 3DP models can be used for surgical trainees’ education inside and outside the operating room [10].

### Limitations

This study has several limitations. The retrospective design inherently introduces selection bias, and the small sample size limits the generalizability of our findings. Not all the included patients have postoperative imaging; this limited the accuracy assessment to fewer patients. Additionally, while our analysis shows improved precision in fracture reduction, it does not account for other potential benefits of VSP, such as a reduced intraoperative decision-making time and enhanced surgical planning efficiency between various surgical specialties and other involved intraoperative technologies. Moreover, this study does not include patient-reported outcomes, which are crucial for a comprehensive evaluation of the impact of VSP on quality of life and patient satisfaction. Even though patients were compared according to fracture type and anatomical distribution, we did not employ a formal injury severity index, such as the Facial Injury Severity Score (FISS), which may have offered a more standardized evaluation of injury complexity. Future research should focus on larger, prospective studies to validate our findings and further explore the cost-effectiveness and long-term benefits of VSP in facial trauma reconstruction. Investigating patient-reported outcomes and quality-of-life measures will provide a more holistic understanding of the impact of VSP on patient care. Additionally, exploring the integration of VSP with other emerging automation tools, such as augmented reality and intraoperative navigation systems, could offer new avenues for enhancing surgical precision and outcomes. We acknowledge these limitations as opportunities for improvement in future prospective studies, which should incorporate standardized severity scoring and matched cohorts to better evaluate the impact of point-of-care virtual planning in facial trauma management.

## 5. Conclusions

VSP integration into facial trauma management offers significant advantages in the management of complex cases. In this study, where VSP and conventional non-VSP approaches were compared, while the VSP group exhibited higher initial preoperative deviations due to more severe and comminuted fracture patterns, pre- and postoperative heatmap analyses showed great postoperative reduction accuracy. When normalized for implant count, the VSP group showed improved surgical efficiency, with shorter operative times and reduced blood loss per implant. These findings support the use of VSP as an effective approach for improving surgical precision and efficiency in complex craniofacial trauma, particularly when addressing complex fracture patterns and multi-specialty surgical interventions.

## Figures and Tables

**Table 1:** Demographics and comorbidities of the study population.

Demographics and Comorbidities		Overall (*n* = 44)	VSP Group (*n* = 23)	Non-VSP Group (*n* = 21)	*p* Value
Age	Age, mean (SD) in years	35.6 (17)	34 (15.7)	37.4 (18)	0.43
Gender	Male (cisgender) (%)	36 (82%)	20 (87%)	16 (76.2%)	0.37
BMI	BMI, mean (SD), kg/m^2^	26.14 (6.5)	24.5 (6.4)	27.9 (6)	0.08
Race and ethnicity	White (non-Hispanic, non-Latino)	36 (81.2%)	18	18	0.14
	Black	2 (4.5%)	_	2	
	Hispanic/Latino	2 (4.5%)	2	_	
	Asian	2 (4.5%)	2	_	
	Other	2 (4.5%)	_	1	
Social Hx ^	Alcohol use (%)	14 (31.8%)	7 (30.4%)	7 (33.3%)	0.84
	Alcoholic drinks/week (SD)		6 (3.6)	4.5 (1.5)	_
	Smoker (%)	15 (34%)	7 (30.4%)	8 (38.1%)	0.6
	Smoking index (pack/year) (SD)	7	382.3 (277.1)	247.5 (123.1)	_
	Drug use	7 (15.9%)	3 (13%)	4 (19%)	0.6
Comorbidities	Hypertension	_	2	1	0.6
	Diabetes mellitus	_	2	2	0.57
	Others: Hyperlipidemia Coronary artery disease	_	4	5	

^ Smoking Methods: cigarettes (10, 76.9%), vaping (E-cigarettes) (2, 15.4%), tobacco gums (5, 38.5%), and tobacco snuff (1, 7.7%). Drug substance use: marijuana, synthetic marijuana, opiates, lysergic acid diethylamide (LSD), and methamphetamines.

**Table 2:** Fracture patterns, patient characteristics, and surgical specialties involved in VSP and non-VSP groups.

Demographics and Comorbidities		Overall (*n* = 44)	VSP Group (*n* = 23)	Non-VSP Group (*n* = 21)	*p* Value
Mechanism of injury (%)	Motor Vehicle Accident (MVA)	14 (31.8%)	10	4	0.09
	Assault	13 (29.5%)	7	6	0.9
	Falls	4 (9.1%)	3	1	0.4
	Sports	6 (13.6%)	2	4	0.3
	Animal Attack	3 (6.8%)	1	2	0.51
	Not mentioned	4 (9.1%)			
Surgical service	Plastic and Reconstructive Surgery	44 (100.0%)	23	21	_
	Oculoplastic Surgery	10 (22.7%)	8	2	0.05 *
	Otolaryngology	7 (15.9%)	7	0	0.01 *
	Neurosurgery	6 (13.6%)	4	2	0.46
	Orthopedic Surgery	5 (11.4%)	4	1	0.19
Reconstruction presentation	Primary (1ry)	40 (91%)	20	20	_
	Secondary (2ry)	4 (9.9%)	3	1	_
Fracture pattern (%)	Naso-orbito-ethmoid (NOE) Complex Fracture	9 (20.5%)	6 (26.1%)	3 (14.3%)	0.34
	Nasal Fracture	4 (9.1%)	1 (0.04%)	3 (14.3%)	0.26
	Zygomaticomaxillary Complex (ZMC) Fracture	20 (45.5%)	10 (43.5%)	10 (47.6%)	0.79
	Orbital Fracture	5 (11.4%)	4 (17.4%)	1 (0.05%)	0.2
	Mandibular Fracture	20 (45.5%)	11 (47.8%)	9 (42.9%)	0.75
	Panfacial ^+^	7 (15.9%)	4	3	0.6
	Frontal Sinus Involvement ^++^	6 (13.6%)	4	2	0.5
Laterality	Unilateral (Rt/Lt)	18 (44%)	8	10	0.48
	Bilateral	23 (56.1%)	13	10	
Le Fort type (if mentioned)	Type I	7 (63.6%)	3	4	0.6
	Type II	7 (63.6%)	4	3	0.8
	Type III	4 (36.4%)	2	2	0.9
Glasgow Coma Scale (GCS)	13 to 15: Mild Traumatic Brain Injury (mTBI)	39 (88.6%)	19	20	0.8
	9 to 12: Moderate TBI	4 (9.1%)	3	1	0.2
	3 to 8: Severe TBI	1 (2.3%)	1	_	_
Abbreviated Injury Scale (AIS) grades	Minor	18 (50.0%)			
	Moderate	10 (27.8%)			
	Severe (Not Life-Threatening)	8 (22.2%)			
	Severe (Life-Threatening, Survival Probable)	_			
Surgery: patient class	Outpatient	27 (61.4%)	12	15	0.3
	Inpatient	17 (38.6%)	11	6	
ASA status	ASA 1	19 (43.2%)	8 (34.8%)	11 (52.4%)	0.17
	ASA 2	16 (36.4%)	8 (34.8%)	8 (38.1%)	
	ASA 3	7 (15.9%)	5 (21.7%)	2 (9.5%)	
	ASA 4	2 (4.5%)	2 (8.7%)	_	

* *p* < 0.05. ^+, ++^ indicate hierarchical subcategories of fracture involvement: ^+^ Panfacial: involving NOE, ZMC, and mandibular fractures. ^++^ indicates Frontal Sinus involvement in NOE fractures.

**Table 3:** Operative metrics between the VSP and non-VSP groups.

	Median (IQR)	SD	*p*-Value
Anesthesia Time (hours): VSP Non-VSP	5.8 (4.1–8.3) 4.6 (3.7–5.6)	3.4 2.7	0.03 *
Procedure Time (hours): VSP Non-VSP	3.8 (2.5–5.6) 2.7 (2.1–3.6)	2.8 2.2	0.03 *
Length of Stay (days): VSP Non-VSP	1.0 (1.0–3.0) 1.0 (0–1.0)	6 4.1	0.3
Blood Loss (cc): VSP Non-VSP	50 (20–200) 50 (20–150)	122.3 78.3	0.7
Follow-Up Duration (days): VSP Non-VSP	65 (34–169) 43 (30–74)	238 356	0.2
Anesthesia Time (minutes)/Implant: VSP Non-VSP	15.4 (11.3–33.8) 19.3 (15.7–31.4)	18.1 16.5	0.4
Procedure Time (minutes)/Implant: VSP Non-VSP	10.5 (6.7–22.5) 10.9 (9–17.7)	11.6 11	0.5
Blood Loss (cc)/Implant: VSP Non-VSP	3.4 (1.7–5.4) 4.1 (2.1–6.4)	3.9 5.1	0.7

SD: Standard Deviation. * *p* < 0.05.

## Data Availability

Research data are available upon request from the Principal Investigator (B.A.S.).

## Abbreviations

The following abbreviations are used in this manuscript:

3D	Three-dimensional
VSP	Virtual surgical planning
3DP	Three-dimensional printing
RMSE	Root mean square error
EPPOCRATIS	Expedited Preoperative Point of Care for Fracture Reduction to Normalized Anatomy and Three-Dimensional Printing to Improve Surgical Outcomes
